# A pilot study on mindfulness based stress reduction for smokers

**DOI:** 10.1186/1472-6882-7-2

**Published:** 2007-01-25

**Authors:** James M Davis, Michael F Fleming, Katherine A Bonus, Timothy B Baker

**Affiliations:** 1 Center for Tobacco Research and Intervention, University of Wisconsin School of Medicine and Public Health, 1930 Monroe St., Suite 200, Madison WI 53711-2027, USA; 2 Department of Family Medicine, University of Wisconsin School of Medicine and Public Health, 777 South Mills St., Madison WI 53715, USA; 3 Health Mindfulness Program, University of Wisconsin School of Medicine and Public Health, 621 Science Dr., Madison, WI53711, USA

## Abstract

**Background:**

Mindfulness means paying attention in the present moment, non-judgmentally, without commentary or decision-making. We report results of a pilot study designed to test the feasibility of using Mindfulness Based Stress Reduction (MBSR) (with minor modifications) as a smoking intervention.

**Methods:**

MBSR instructors provided instructions in mindfulness in eight weekly group sessions. Subjects attempted smoking cessation during week seven without pharmacotherapy. Smoking abstinence was tested six weeks after the smoking quit day with carbon monoxide breath test and 7-day smoking calendars. Questionnaires were administered to evaluate changes in stress and affective distress.

**Results:**

18 subjects enrolled in the intervention with an average smoking history of 19.9 cigarettes per day for 26.4 years. At the 6-week post-quit visit, 10 of 18 subjects (56%) achieved biologically confirmed 7-day point-prevalent smoking abstinence. Compliance with meditation was positively associated with smoking abstinence and decreases in stress and affective distress.

**Discussions and conclusion:**

The results of this study suggest that mindfulness training may show promise for smoking cessation and warrants additional study in a larger comparative trial.

## 1. Background

Mindfulness may be characterized as paying attention to the moment-to-moment unfolding of internal experience such as physical sensation, emotion, craving, agitation, etc; while maintaining a mental posture of acceptance toward each experience as a passing phenomenon without acting on it, judging it, ruminating about it, or trying to fix it [1-3]. Mindfulness is a skill, and like learning a musical instrument, is developed through repeated daily practice. An intended result of mindfulness practice is that a mental orientation of mindfulness will develop toward daily events providing enhanced mental/emotional flexibility and clarity to deepening one's enjoyment of life and making one more skillful in facing life's challenges.

The secular instruction of mindfulness has been formulated through Mindfulness Based Stress Reduction (MBSR), a stress reduction program developed by Jon Kabat-Zinn, now taught in over 240 institutions worldwide [4]. MBSR is structured to provide basic instructions in mindfulness and in starting a daily meditation practice to individuals, most of whom have not had prior exposure to the subject. MBSR requires participants to meet as a group for 2–3 hours, once per week, for eight weeks, meet for a 7-hour "day of mindfulness", and practice mindfulness meditation 45 minutes per day, six days per week.

One motivation for developing a study to evaluate the effect of mindfulness training on smoking cessation is that mindfulness training has been shown to decreases stress, a trigger for tobacco use. Stress has been strongly correlated with smoking rate and relapse rate through studies evaluating financial stress [4], social stress [5], and stress of job loss [5]. Studies on mindfulness training have demonstrated a reduction in stress related symptoms [6], and reductions in salivary cortisol (a biomarker for stress) [12,21].

Another motivation for evaluating the effect of mindfulness on smoking cessation is that mindfulness training has been shown to decreases negative affect, also associated with drug use behavior. Mounting studies support the idea that negative affect is a potent stimulus for drug-seeking behavior and smoking relapse [7-10]. Several studies have shown mindfulness training to be associated with a reduction in affective distress of mood disturbances [11-14]. Other studies have shown an association between mindfulness training and improved sense of well-being [15-17] as well as mindfulness and decreased depressive relapse [18-20].

A mechanism postulated for the use of mindfulness in smokers is that mindfulness might be used as a cognitive skill for managing craving, withdrawal symptoms and emotional distress, and that this skill might persist through time exerting a long-term effect on response to smoking cues. The pilot study had a more preliminary scope of inquiry, to test feasibility and determine if additional study is warranted.

## 2. Methods

### 2.1. Recruitment

Subjects were recruited from the Dane County, Wisconsin, region, using a pre-existing smoking study recruitment phone list managed through the University of Wisconsin School of Medicine and Public Health, Center for Tobacco Cessation and Intervention (UW-CTRI). Advertising used the statement "Quit Smoking Study" without reference to meditation. Subjects from this phone list had been excluded from a previous study because they a) reported that they would not remain in the region for 3 years (n = 10), b) reported a recent history of anxiety, depression or bipolar disorder (n = 5), or c) reported past, but not current use of Bupropion (n = 4). Inclusion criteria required subjects to be 18 or over and smoke 10 or more cigarettes per day. No exclusion criteria were used, though incidentally no subjects used NRT or Bupropion during the study. Twenty-two potential subjects attended a one-hour orientation describing the intensive nature and time requirements of the intervention. Of these, 18 subjects signed the consent form and attended the first class. The other four excluded themselves after the orientation, reporting "scheduling" or "transportation" constraints. The study protocol received approval from, and followed procedures in accord with, the standards of the University of Wisconsin Health Sciences Institutional Review Board.

### 2.2. Intervention

MBSR was taught to participants in the usual manner by instructors at the UW Health Mindfulness Program without alteration of the central course content or requirements. As a smoking intervention, there were some differences from a typical MBSR course: subjects entered the intervention with the express purpose of smoking cessation; a quit date was provided, addiction questionnaires and carbon monoxide testing was performed at each weekly meeting, and course instructions were focused at times on how one might apply mindfulness to craving and withdrawal symptoms. In the spirit of non-goal-directedness central to mindfulness training, subjects were encouraged not to focus on smoking cessation as a "goal" for the intervention, but instead, to direct their intention toward developing a mindful orientation toward their lives. Subjects were encouraged to apply moment-to-moment non-judgmental awareness to strong emotions or thoughts, which in this case often involved craving, negative affect, or withdrawal symptoms. Subjects were encouraged to practice mindfulness throughout their day, including during meals, social interactions, and moments associated with situational drug use threats. The quit-smoking day was on the 7-hour "day of mindfulness", ten days prior to the end of the intervention. Considerable testing was performed at the final meeting. No pharmacotherapy was used.

### 2.3. Outcome measures

Measures of meditation compliance, smoking, and stress were taken at each meeting, including one day, eight days and six weeks post quit. Compliance with meditation was tested via 7-day meditation calendars provided weekly. Smoking abstinence was tested via 7-day smoking calendars and verified via carbon monoxide breath test (abstinence defined as a carbon monoxide level under10 ppm) [22,23]. Questionnaires used to test changes in reported stress and affective distress included respectively, the Perceived Stress Scale (PSS) and the Symptoms Check List (SCL-90-R). The Perceived Stress Scale is a questionnaire designed to provide an assessment of symptoms of stress over the previous week. The PSS has robust reliability and validity [34] and has been used in multiple studies to measure the effect of mindfulness training on stress [18-21]. SCL-90-R is a 90 item questionnaire designed to test self-reported affective distress associated with nine categories: Somatization, Obsessive Compulsive Disorder, Interpersonal Sensitivity, Depression, Anxiety, Hostility, Phobic Anxiety, Paranoid Ideation and Psychoticism. The SCL-90-R has been used in numerous studies on mindfulness [11,13,19], and reliability and validity has been tested in multiple populations [24-31].

.

## 3. Results

### 3.1. Sample characteristics

The sample (*n *= 18) had an average age of 45.2 years (range: 22 – 67), was composed of 10 women and 8 men, all Caucasian. The average cigarettes smoked per day was 19.9 (range: 10 – 40), and average number of years smoked was 26.4 (range: 4 – 44). Subjects were not excluded based on motivation to quit smoking, however, during recruitment all potential subjects in the study reported their motivation to quit smoking as "high". Subjects reported no use of tobacco related pharmacotherapy.

### 3.2. Compliance with daily meditation

Subjects were provided with an audio-CD with a 45 minute guided meditation, asked to use the CD once a day, six days a week (270 minutes per week) and perform additional meditation if possible. Subjects were asked to record the number of minutes of meditation practiced each day in a logbook and were strongly encouraged to be consistent and honest in their daily reporting. Of the 18 subjects that completed the study, 3 patterns of compliance were evident: 1) those meditating equal to or more than the requested minimum (270 minutes per week) were defined as "highly compliant meditators"; 2) those who meditated less than the requested minimum were defined as "moderately compliant meditators"; and 3) those who stopped meditating within the first 4 weeks of the intervention were defined as "non-compliant meditators". Highly compliant meditators (n = 8) meditated a mean of 51.8 min/day (SD = 12.6 min/day); moderately compliant meditators (n = 5) meditated mean of 23.4 min/day (SD = 6.1 min/day), and non-compliant meditators (n = 5) meditated mean of 11.3 min/day (SD = 5.2 min/day). Differences in meditation time between groups were primarily the effect of missed days of meditation, though meditations under 45 minutes played a small role as well.

### 3.3. Attrition

All highly and moderately compliant meditators remained in the intervention. All non-compliant meditators (n = 5) dropped out of the intervention before the quit date. Three of the five non-compliant meditators dropped out during the 2^nd ^week, the other two dropped out during the 4^th ^week. Follow-up phone calls revealed the following reasons for dropout: "conflicts with summer vacation scheduling"(n = 3); "not enough time for daily meditation" (n = 1); and "meditation is too hard" (n = 1). Because these subjects dropped out, post-quit data was not available from carbon monoxide testing, smoking logbooks, and written instruments. Phone interviews at the 6-week post-quit follow-up revealed that none who dropped out stopped smoking on their own.

### 3.4. Smoking abstinence at the 6-week follow-up visit

Smoking abstinence was tested via weekly 7-day smoking calendars and confirmed via weekly carbon monoxide (CO) breath tests. There were no discrepancies between self-reported abstinence and CO-level defined abstinence. Of the 18 subjects who entered the intervention, ten (55.6%) (95% CI = 32.7–78.5) attained point-prevalent abstinence six weeks after the quit date. Of the eight subjects who failed to attain abstinence, five failed due to dropout before the quit date, while three quit but relapsed before the 6-week follow up.

### 3.5. Predictors of smoking abstinence

Post-hoc analysis was performed to identify differences between subjects who did and did not achieve biologically confirmed 6-week point prevalent smoking abstinence. Factors that predicted abstinence were time in meditation, interest in meditation and affective distress. Abstinent subjects (n = 10) meditated a mean 45.3 (SD = 15.8) minutes per day vs. non-abstinent subjects (n = 3) (excluding dropouts) who meditated a mean of 22.4 (SD = 5.2) minutes per day. The difference for time in meditation between groups was significant by two-sample *t*-test (t_(11) _= 2.4, p = .03). Inclusion of dropouts in this calculation increased the magnitude and significance of the difference between groups. Reporting a "strong interest" in meditation significantly predicted smoking abstinence by two-proportion z test (abstinent 80% vs. non-abstinent 25%; z = 2.33, p < .01). Finally, greater baseline affective distress predicted smoking abstinence (pooled two-sample *t*-test: t_(10) _= 2.4, p = .04). Abstinent subjects had a mean baseline SCL-90-R score of 89.9 (SD = 30.1; one incomplete test result was omitted) while non-abstinent subjects had a mean score of 57.8 (SD = 30.4).

### 3.6. Symptoms of stress in moderately vs. highly compliant meditators

**Figure 1:**
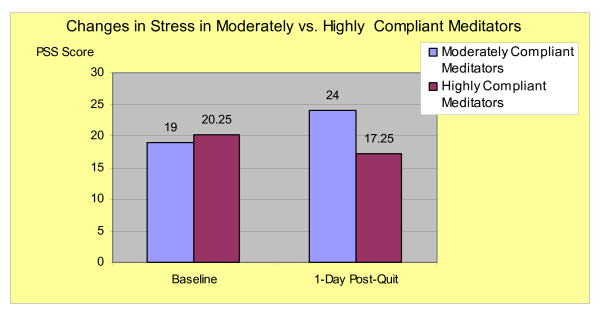
Perceived Stress after Quitting Smoking in Moderately vs. Highly Compliant Meditators.

Moderately vs. highly compliant meditators were compared with regard to symptoms of stress (PSS) at baseline and at 1-day post-quit (highest post-quit PSS scores for all groups). Moderately compliant meditators showed an increase of mean PSS scores from 19 (baseline) to 24 (1-day post-quit) compared to highly compliant meditators who showed a decrease in mean PSS scores from 20.25 (baseline) to 17.25 (1-day post-quit) (Figure-1). The difference in change of PSS scores between the two groups was significant with *t*-test for equal variance: t_(11) _= -3.11, (p < .01) (Levenes's test showed similar variance). As with most pilot studies, outliers with a small sample size may have contributed to the significance.

### 3.7. symptoms of affective distress in moderately vs. highly compliant meditators

**Figure 2:**
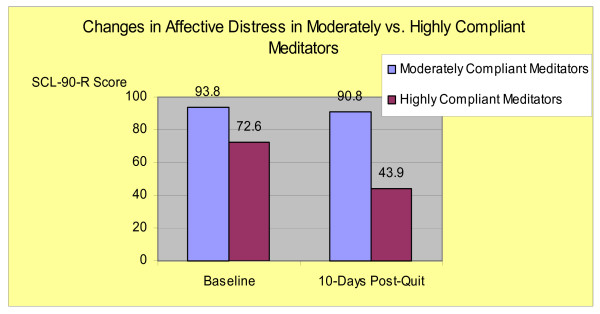
Changes in Affective Distress in Moderately vs. Highly Compliant Meditators.

Symptoms of affective distress (SCL-90-R) were assessed at two time points only: baseline and the 8-day post-quit visit. Moderately compliant meditators showed a decrease in mean SCL-90-R scores from 93.8 (baseline) to 90.8 (10-days post-quit) compared to highly compliant meditators who decreased from 72.6 (baseline) to 43.9 (10-days post-quit) (Figure-2). The difference in change of mean SCL-90-R scores between the two groups was 25.7 points but not significant with *t*-test for equal variance: t_(11) _= -1.38, (p < .19) (Levenes's test showed similar variance).

### 3.8. Subject acceptance

During the final meeting (10-days post-quit) subjects completed a course evaluation. This provided an opportunity for subjects to make recommendations for future devolvement of the intervention. The course evaluation contained open-ended questions as well as directed questions regarding perceived strengths and weaknesses of the intervention. Representative responses to the open-ended question, "What did you like or dislike about the course?" included:

• "I never thought that I would enjoy meditation but I found that I do it every day. Quitting smoking is only one of many benefits of mindfulness."

• "Everyone was wonderful. I feel empowered for the first time in years. I've decided to get a better job."

• "I am sleeping better and I have more energy."

Responses to directed questions provided more critical feedback and included the following:

• "Was there was enough time spent on exercises for smoking cessation?" Four said "no".

• "Did the course end too soon after quit date?" Ten said "yes".

• "Did you need more help with withdrawal symptoms?" Six said "yes".

## 4. Discussions and conclusion

### 4.1. Abstinence

A 6-week abstinence rate of 56% is relatively high when compared to results of a previous comparable study population showing 33% point prevalent 6-week post-quit abstinence rate in a when provided with moderately intensive counseling, no pharmacotherapy, and seen weekly at the time of testing [33]. It is noteworthy that ten of the thirteen subjects who attempted to quit (did not drop out) achieved abstinence at six weeks. The best explanation for this may be that MBSR is an intensive intervention and that due to its intensive nature, dropout rates are high but success rates in those who complete the intervention is also high. Although there were considerable limitations to this pilot study, this relatively high abstinence rate suggests that mindfulness training may have some promise as an intervention for smoking cessation.

### 4.2. Meditation compliance as a possible predictor of smoking abstinence

In this sample, compliance with meditation practice appeared to be associated with smoking abstinence. The point-prevalent 6-week abstinence rate was 100% among highly compliant meditators, 40% among moderately compliant meditators and 0% among non-compliant meditators (dropouts). One conclusion from this data would be that compliance with meditation is a trait of smokers who are motivated to quit and that meditation itself does not confer a therapeutic effect. Results from baseline questionnaires on motivation to quit smoking, however, do not support this: all subjects reported a "high-level of motivation" to quit smoking. Even if meditation time did co-vary with motivation to quit, the association between meditation time and smoking abstinence makes it difficult to dismiss the possibility that meditation provided some effect over and above motivation alone.

### 4.3. Interest in learning meditation may predict smoking abstinence

Subjects with a baseline report of a "strong interest in learning meditation" had a significantly higher 6-week point prevalent abstinence. The further finding that eight of the ten who achieved abstinence also reported a strong interest in meditation at baseline, suggests that interest in meditation and compliance with meditation may be related. The findings suggest that the intervention may be most effect for smokers with an interest in learning meditation.

### 4.4 Affective distress may predict smoking abstinence

Subjects with higher SCL-90-R scores appeared to achieve higher levels of 6-week point prevalent abstinence. It appears to be counter-intuitive that subjects with higher levels of affective distress would achieve higher levels of abstinence. If however, we consider the possibility that the intervention may provide relief from affective distress, subjects with higher affective distress may achieve more benefits from mindfulness, and so are more motivated to practice.

### 4.5. Changes in reported stress and affective distress

The finding that highly compliant meditators demonstrated substantial decreases in reported stress and affective distress and attained 100% abstinence suggests the possibility that reduction in stress or affective distress may be a therapeutic mechanism by which mindfulness training has its potential effect. A decrease in stress in highly compliant meditators after quitting smoking is not a typical finding for smoking cessation interventions and suggests that meditation may exert an effect on outcomes though stress reduction. Another finding in the study was that highly compliant compared to non-compliant meditators demonstrated a drop in SCL-90-R scores. The difference, however, was confounded in this small sample by a large variance and did not reach statistical significance. As such, it is unclear from these study results whether compliance with meditation in this sample is associated with decreased affective distress.

### 4.6. Study limitations

A principal limitation to the intervention's effectiveness is the omission of pharmacotherapy. Pharmacotherapy was purposefully excluded from the study so as to allow for the appraisal of mindfulness training as a smoking intervention without the confounding therapeutic effects of medication. Limitations of the study also include a small sample size, absence of a control group and short-term follow-up. Since this was a pilot study aimed at assessing feasibility, pre-post study design was used and comparisons were made post-hoc based on compliance with meditation practice. Additionally, the program orientation, which discouraged four individuals from participating, may have introduced a selection bias that would lead to an overestimation of the intervention efficacy in other populations. Subsequent testing of mindfulness training as a smoking intervention would include the use of pharmacotherapy, random assignment of smokers to the intervention vs. control group, and six-month follow-up.

### 4.7. Attrition

Attrition rate was five of eighteen subjects (28%) with three dropouts in the first two weeks. Exit interviews with dropouts suggest that subjects left the study primarily because they were unaware of the high time commitment or unaware of the nature of the intervention (meditation practice). Thus, it is possible that the attrition rate would decrease if the nature and intensity of the intervention were made clearer to prospective subjects.

### 4.8. Compliance with meditation practice

There appeared to be a high level of compliance with meditation among those in the study. Even those later categorized as "moderately compliant meditators" meditated on average 23.4 minutes per day. Notably, recruitment flyers advertised only a "quit smoking study" with no mention of meditation and no subjects were excluded based on motivation. It could be that our small sample was uncharacteristically motivated to learn meditation or may be due to the fact that highly skilled veteran mindfulness instructors who are skilled at motivating mindfulness practice.

### 4.9. Further study

A majority of the sample recruited expressed a "strong interest" in meditation, a characteristic most likely not reflected in the population at large. The intensive training program might be cost-effective for treatment-resistant smokers who are unable to quit with less intensive interventions. Given the rather high abstinence rates in this small sample, and relative increased effectiveness in subjects with higher levels of affective distress, mindfulness training may hold promise in select and challenging populations. From the results available, a larger randomized comparative trial appears to be warranted.

## Abbreviations

Mindfulness Based Stress Reduction (MBSR)

University of Wisconsin School of Medicine and Public Health, Center for Tobacco

Cessation and Intervention (UW-CTRI).

## Competing interests

The author(s) declare that they have no competing interests.

## Authors' contributions

JM Davis designed the study, recruited subjects, performed data collection, analysis and manuscript writing. MF Fleming and TB Baker provided oversight in study design, interpretations of results and manuscript writing. KA Bonus was a full collaborator in the intervention design and directed intervention implementation. All authors read and approved the final manuscript.

## Pre-publication history

The pre-publication history for this paper can be accessed here:


